# Evaluation of Women's Awareness and Knowledge of Planned Oocyte Cryopreservation at Different Sociocultural Levels: A Cross-Sectional Survey

**DOI:** 10.1155/ogi/8491436

**Published:** 2025-03-03

**Authors:** Sara Salihi, Fatma Başak Tanoğlu, Ali Gökçe, Hikmet Tunç Timur, Özge Pasin, Pınar Özcan

**Affiliations:** ^1^Faculty of Medicine, Department of Obstetrics and Gynecology, Bezmialem University, Istanbul, Türkiye; ^2^Yenimahalle Training and Research Hospital, Department of Obstetrics and Gynecology, Yildirim Beyazit University, Ankara, Türkiye; ^3^Department of Obstetrics and Gynecology, Dokuz Eylül University School of Medicine, Izmir, Türkiye; ^4^Faculty of Medicine, Department of Biostatistics, Bezmialem University, Istanbul, Türkiye

**Keywords:** age-related infertility, planned oocyte freezing, social oocyte freezing

## Abstract

**Objective:** This study evaluated awareness and knowledge of planned oocyte cryopreservation (POC) among Turkish women across diverse sociocultural backgrounds.

**Design:** This is a cross-sectional survey with a 56-item self-administered electronic questionnaire.

**Setting:** Istanbul, Turkiye, June to September 2022.

**Population:** The study evaluated 915 participants between 21 and 45 years.

**Methods:** Women completed a 56-item self-administered online electronic questionnaire survey to identify their demographic information, perspective on family planning, future fertility expectations/plans, oocyte freezing, and degree of knowledge.

**Main Outcome Measures:** Comparison of participants considering and not considering POC.

**Results:** A total of 464 (50.7%) women indicated an intent to undergo POC in the future, with statistically significantly higher university graduates in the group clearly considering POC (*p*=0.044) and a higher rate of singles indicating an intent to POC. A total of 546 (59.7%) women planned to have children in the future; the participants considered the age of 39.37 (±5.01) as “old” for pregnancy; 748 (81.7%) of the participants thought that the information about age-related fertility decline and POC should be a part of the annual gynecological examination. The level of knowledge about family planning/postponing fertility and POC between the two groups showed that the group considering POC provided significantly more accurate responses to seven items (5th, 6th, 7th, 10th, 11th, 14th, and 17th questions). The group not considering POC answered correctly, “What is the rate of spontaneous female conception?” (*p*=0.047).

**Conclusion:** The target population expected to benefit from POC in our country had low knowledge and awareness of age-related fertility decline and POC. Most women expected the information to be a part of the annual gynecological examination. The main determinants of the knowledge score and considering POC are education level and relationship status.

## 1. Introduction

In recent decades, society has postponed the beginning of parenthood due to lifestyle and socioeconomic changes, such as women's education, job redundancy, family care responsibilities, economic concerns, the lack of a partner, and the desire to establish a business [1]. These shifts have allowed for greater autonomy, but it is very well known that there is a strong relationship between female age and fertility. Evidence clearly defines that female fecundity sharply declines with age [2]. Therefore, women commonly face age-related infertility by postponing parenthood. Hutterites, a well-documented epidemiological cohort, did not practice contraception and exhibited high fertility, averaging 9.6 children per woman with a low infertility rate of 2.4%. However, their fecundity declined steeply after age 35, with a substantial proportion ceasing reproduction by ages 40 and 45 [3].

Oocyte cryopreservation (OC) is a marvelous milestone in assisted reproductive technologies (ART). The first successful pregnancy with OC was achieved in 1986 [4]. OC became widely accessible after the American Society for Reproductive Medicine (ASRM) declared in 2013 that OC was not an experimental procedure [5]. Moreover, by 2017, the ASRM deemed that OC was morally permissible for safeguarding against age-related infertility [6].

Various terms such as social oocyte freezing and elective oocyte freezing have been used in the literature to describe OC due to postponing childbearing. Additionally, British researchers have proposed the term “OC for anticipated gamete exhaustion” or “AGE” [7]. However, the ASRM recommends the use of “planned oocyte cryopreservation” (POC) for procedures at nonemergency fertility preservation [6].

Although POC has become a viable option for patients and gynecologists for nonemergency fertility preservation, its success depends on the primary determinants, including age at the time of OC and the number of oocytes stored [8–10]. Recent publications have recommended that planned OC and embryo transfer could be completed before 40 years to maximize the success rates of procedure [11]. Walker et al. clearly showed no successful pregnancies in women aged 40 or older in a review, which included the results of 921 cases [12]. Furthermore, it should be kept in mind that maternal morbidity and mortality and neonatal health risks also rise with maternal age or conceived through ART [13]. OC, an invasive procedure, also carries risks, including the invasive nature of oocyte retrieval, theoretical risks of infectious disease transmission, and complications from delayed use of frozen oocytes [14]. Patients must be informed that OC does not halt age-related fertility decline or guarantee successful conception [15].

International studies in many countries, including Australia, Italy, the UK, and Canada, have evaluated the knowledge and attitude of POC [16–19]. Studies on the awareness and perspectives of POC revealed different levels of interest and understanding of POC. To the best of our knowledge, no study has addressed these concerns in Türkiye. This study evaluated awareness and knowledge of POC among Turkish women across diverse sociocultural backgrounds.

## 2. Methods

### 2.1. Study Design, Participants, and Setting

We conducted a cross-sectional survey study from June to September 2022 in Istanbul. The survey was administered independently and voluntarily with a 56-item, self-administered electronic questionnaire to identify demographic information, perspective on family planning, future fertility expectations/plans, oocyte freezing, and degree of knowledge. The participants had access to the survey via social media, which enabled a countrywide setting and the inclusion of diverse socioeconomic backgrounds. Due to the lack of internationally validated surveys, we developed our survey based on previous related research. The electronic questionnaire was completed by 915 women between 21 and 45 years. The age groups were defined according to a study by Milman et al. [20]. The recruitment was terminated on achieving the minimum requirement of 829 (with a 10% dropout, 912 participants were required; 915 women participated while fulfilling the inclusion criteria). This IRB-approved study was accepted by the Ethics Committee of Bezmialem University (Ethics Board No.: E-54022451-050.05.04-65376).

### 2.2. Statistical Analysis

Sample size and power calculation determined that sufficient statistical power required a minimum of 829 participants to achieve a 10% frequency (1 in 10 response rate), with a 95% confidence level and a 2% margin of error (power = 0.80, Type 1 error = 0.05, and Type 2 error = 0.20) (Milman et al. [20]). Descriptive statistics for the qualitative variables in the study were presented as numbers and percentages; descriptive statistics for the quantitative variables were provided as means, medians, standard deviations, minimums, and maximums. The normality of the numerical variables in the study was assessed using the Shapiro–Wilk test. The independent *t*-test (Student's *t*-test) was used to compare the means of groups consisting of two categories; the Mann–Whitney *U*-test was used to compare medians. One-way analysis of variance (ANOVA) was used to compare the means of groups with more than two categories, and pairwise comparisons were performed using the Tukey test. The Kruskal–Wallis test was used to compare the medians of groups with more than two categories, with multiple comparisons conducted using the Dunn test. Relationships between categorical variables were analyzed using the Pearson chi-square test. A significance level of 0.05 was considered statistically significant, and calculations were performed using SPSS Version 28 (IBM SPSS Statistics for Windows, Armonk, NY: IBM Corp, 2021).

## 3. Results

A total of 915 women participated in our survey and completed the questionnaire. An additional 350 individuals also used the online survey; however, they were excluded due to being male or undisclosed gender (*n* = 76), undisclosed age (*n* = 104), and failure to complete the survey (*any unanswered questions*) (*n* = 170). Subsequent findings were reviewed. Further details on participant demographics, including age, education, and marital status, are provided in Table 1.

A total of 464 (50.7%) women indicated an intent to undergo POC in the future, whereas 451 (49.3%) showed disinterest. A comparison of educational levels, age, relationship status, and income levels showed no significant difference in income levels and age between the groups (Table 1), with statistically significantly higher university graduates in the group considering POC; high school graduates were higher in the group not planning POC (*p*=0.044) (Table 1). Analyzing the relationship status showed that singles had a higher rate of indicating an intent to undergo POC (Table 1).

A total of 546 (59.7%) women planned to have children in the future; the participants considered the age of 39.37 (±5.01) as “old” for pregnancy (Table 2). Additionally, 748 (81.7%) of the participants thought that the information about age-related fertility decline should be a part of the annual gynecological examination; 741 (80.9%) respondents believed that the information about POC should also be a part of the annual gynecological examination (Table 3); 223 (18.2%) in our cohort were positive about donating to an anonymous egg donor program if they decided not to use their eggs (Table 3).

The comparison of the results of 19 questions to measure the level of knowledge about family planning/postponing fertility and POC between the two groups showed that the group considering POC provided significantly more accurate responses to seven items (5th, 6th, 7th, 10th, 11th, 14th, and 17th questions in Table 4). The group not considering POC answered correctly, “What is the rate of spontaneous female conception?” (*p*=0.047) (Table 4). There was no significant difference in response accuracy for the remaining 11 information items related to fertility with older ages and POC between the two groups (Table 4).

## 4. Discussion

To the best of our knowledge, in the light of literature, this is the first study with the largest cohort in the Turkish community to evaluate awareness and knowledge of POC among women across diverse sociocultural backgrounds. Our results showed that 59.7% of women of reproductive age planned to have children in the future, and 50.7% of women indicated an intent to undergo POC in the future. The tendency to consider POC was higher in single women with high sociocultural levels. The results of a cross-sectional study on 422 women between 18 and 38 years in the UAE demonstrated that education level was the main determinant of the knowledge of POC, and the cost was the main barrier for POC [21]. Of our cohort, 25.4% were married. Milman et al. also reported that being single was more strongly associated with considering POC than being married [20]. Although nonfreezers were significantly older than potential freezers in the study by Stoop et al. [22], there was no difference in age or income level between the two groups in our study.

Our results also showed that most women expected that information about age-related fertility decline and POC should be a part of the annual gynecological examination. Also, considering the rate of correct answers to the questions evaluating the level of knowledge of POC between groups considering POC and not considering POC, the group considering POC had statistically significantly higher rates of correct answers in seven questions than the others. These questions were particularly on age related to fertility decline and POC. This difference may be attributed to the fact that the group planning to undergo POC may have performed more research on the subject or was more attentive to their prior knowledge due to selective perception. The rates of correct responses for family planning, age-related fertility decline, and POC revealed that the target population expected to benefit from POC had low knowledge and awareness. Therefore, state and health authorities should inform society and increase awareness so that the population probably affected by age-related fertility decline can benefit from existing technology.

Studies on the awareness and perspectives of POC revealed different levels of interest and knowledge regarding POC. This discrepancy could be linked to cultural, religious, and chronological variables among the cohorts. In Western contexts, according to the results of a survey including 1914 women between 21 and 40 years in Belgium, 31.5% of women defined themselves as potential social oocyte freezers, of which 3.1% considered the procedure [22]; a 2017 study by Milman et al. in an American cohort reported that 21.6% women considered POC [20]. A cross-sectional survey involving 1000 women between 21 and 45 years in the United States showed that 87.2% were aware of POC; however, only 25% considered it viable, reflecting barriers such as cost and cultural attitudes [20]. In Austria, despite legal restrictions, women showed positive attitudes toward POC [23]. Patients in the United Kingdom expressed high satisfaction with their decision to cryopreserve oocytes [24].

In non-Western contexts, POC awareness remains underexplored. A study in Iran found that female university students had positive attitudes toward POC with a lack of knowledge of age-related infertility and POC [25]. Financial barriers also influence attitudes. Sayegh et al. evaluated the knowledge and attitude of women between 18 and 38 years about POC in the United Arab Emirates in a cross-sectional study. They showed that approximately 91% of women already knew about POC [21]. They highlighted two important barriers, including education level and cost, for POC. Our group had a higher percentage of women who intended POC in the future (49.3%). However, a prospective descriptive study evaluated the significance of government assistance on POC in local communities and suggested that educational and financial assistance was required to offer this service to the women who need it [26]. Kikuchi et al. also highlighted “social infertility” in Japan and emphasized the need to assist women whose fertility was at risk with the fertility preservation method [27].

This study has a few limitations. Our cohort had a higher number of married participants, and we did not evaluate embryo freezing as another option for fertility preservation in married women. Embryo freezing also provides an opportunity for preimplantation genetic screening, especially in advanced maternal age. Therefore, using embryos may lead to higher pregnancy and live birth rates. In some regions, in vitro fertilization may be covered by insurance; thus, married women may prefer it to embryo freezing. Future studies should focus on single women to prevent selection bias. Another limitation pertains to social issues. Sexual orientation is a significant factor in OC. For example, the transgender community is a potential candidate for the procedure [28]; however, the topic remains relatively underdiscussed in our country. Therefore, the results cannot be generalized to other populations or cultures.

Finally, the crucial limitation of our study is the absence of a standardized questionnaire, which could raise concerns regarding the validity and reliability of the questionnaire. We tried to include all questions to identify demographic information, perspective on family planning, future fertility expectations/plans, OC, and degree of knowledge due to the lack of internationally validated surveys; thus, the survey was based on previous related research.

## 5. Conclusion

The target population expected to benefit from POC in our country had low knowledge and awareness of age-related fertility decline and POC. Most women wanted the information to be a part of the annual gynecological examination. The main determinants of the knowledge score and considering POC were education level and relationship status. Currently, ART are partially funded by national health insurance; however, OC is not funded by health insurance, and it can be performed only for emergency fertility preservation in our country. The donation of cryopreserved oocytes is also not legal. This research may contribute to a deeper understanding of how demographic, cultural, public health policies, and economic factors shape attitudes toward POC and improve access to fertility preservation technologies. Additional studies are required to investigate people's perspectives on the topic, determine the characteristics of the target population expected to benefit from POC, and increase public knowledge and awareness. We believe that our research will be an essential step in shedding light on this topic.

## Figures and Tables

**Table 1:** Demographic characteristics of the women.

Variables	All women (*N* = 915), *n* (%)	Not considering oocyte freezing (*N* = 464), *n* (%)	Considering oocyte freezing (*N* = 451), *n* (%)	*p*
Age (years)				0.776
21–26	595 (65)	300 (64.6)	295 (65.4)	
27–32	193 (21.1)	95 (20.4)	98 (21.7)	
33–38	67 (7.3)	35 (7.5)	32 (7)	
39–45	60 (6.6)	34 (7.3)	26 (5.7)	
Level of education				0.044
Primary school graduate	21 (2.3)	11 (2.3)	10 (2.2)	
High school graduate	79 (8.6)	52 (11.2)	27 (5.9)⁣^∗^	
University graduate	658 (71.9)	322 (69.3)	336 (74.5)⁣^∗^	
Graduate	157 (17.2)	79 (17)	78 (17.2)	
Monthly income level (TL)				0.214
1000–2000	281 (30.7)	153 (32.9)	128 (28.3)	
2000–5000	149 (16.3)	81 (17.4)	68 (15)	
5000–10000	283 (30.9)	135 (29)	148 (32.8)	
> 10,000	202 (22.1)	95 (20.4)	107 (23.7)	
Relationship status				0.021
Married	234 (25.6)	138 (29.7)	96 (21.2)⁣^∗^	
Divorced	11 (1.2)	5 (1)	6 (1.3)	
Engaged	24 (2.6)	9 (1.9)	15 (3.3)	
Single/Other	646 (70.6)	312 (67.2)	334 (74)⁣^∗^	

⁣^∗^Significant group difference at post hoc analysis that contribute to overall hypothesis test significance.

**Table 2:** Questions about family planning and postponing childbearing.

	All females (*n* = 915)
Do you plan to have children in the future?	
Yes, *n* (%)	546 (59.7)
No, *n* (%)	150 (16.4)
Undecided, *n* (%)	219 (23.9)
At what age do you plan to have your first child? (mean ± SD)	28.31 ± 3.37 (16–44)
At what age do you think you will have your first child? (mean ± SD)	29.89 ± 3.26 (20–45)
At what age do you think you will be old for pregnancy? (mean ± SD)	39.37 ± 5.01 (22–65)
What is the average age of first childbearing for women in Turkey? (mean ± SD)	24.38 ± 3.41 (10–48)
What is the percentage of not having children for women pursuing an academic career? (mean ± SD)	43.65 ± 20.61 (0–100)
What is the fertility of women at different ages?	
Fertility declines over the age of 30; *n* (%)	99 (10.8)
Fertility declines over the age of 35; *n* (%)	385 (42.1)
Fertility declines over the age of 40; *n* (%)	431 (47.1)
What is a woman's chance of spontaneous pregnancy?	
%20–25; *n* (%)	189 (20.7)
%25–30; *n* (%)	159 (17.4)
%30–35; *n* (%)	231 (25.2)
%35–40; *n* (%)	93 (10.2)
> %40; *n* (%)	243 (26.6)
What is a woman's percent chance of conceiving with IVF?	
%20–25; *n* (%)	146 (16)
%25–30; *n* (%)	163 (17.8)
%30–35; *n* (%)	223 (24.4)
%35–40; *n* (%)	124 (13.6)
> %40; *n* (%)	259 (28.3)
What is a woman's percent chance of conceiving with frozen oocytes?	
%20–25; *n* (%)	223 (24.4)
%25–30; *n* (%)	184 (20.1)
%30–35; *n* (%)	226 (24.7)
%35–40; *n* (%)	93 (10.2)
> %40; *n* (%)	189 (20.7)

**Table 3:** Questions about planned oocyte cryopreservation.

	All females *n* (%)
1. Have you heard of social egg freezing before?	
Yes, I/my partner had the egg freezing procedure.	12 (1.3)
Yes, I know about social egg freezing.	259 (28.3)
Yes, but I have no information.	438 (47.9)
No.	196 (21.4)
2. If so, where did you hear it?	
Internet	209 (22.8)
Social media	123 (13.4)
Friends and environment	192 (21)
Other	223 (24.4)
3. Would you consider freezing oocyte to become a future mother for nonmedical reasons at some point in your life?	
Yes	451 (49.3)
No	464 (50.7)
4. Which of the following reasons in the literature justifying social oocyte freezing do you agree with?	
Letting a woman postpone motherhood for work commitments and professional opportunities	489 (19.8)
Allowing a woman to take care of other people (care commitments)	207 (8.4)
Letting a woman be ready for motherhood	639 (25.9)
Allowing a woman to achieve her economic stability	496 (20.1)
Letting a woman find the “right” partner	540 (21.8)
None of the above	99 (4)
5. At what age would you consider freezing oocyte for nonmedical reasons?	
20–30	222 (24.3)
30–35	282 (30.8)
35–40	113 (12.3)
> 40	158 (17.3)
6. Which age group is most suitable for oocyte freezing for nonmedical reasons?	
20–30	453 (49.4)
30–35	265 (29)
35–40	113 (12.3)
> 40	84 (9.2)
7. At what age do women have a slight decrease in their ability to conceive?	
30–35	273 (29.8)
35–40	495 (54.1)
> 40	147 (16.1)
8. At what age do women have a marked decline in their ability to conceive?	
30–35	20 (2.2)
35–40	205 (22.4)
> 40	690 (75.4)
9. For women over 30, overall health and fitness is a better indicator of fertility than age.	
Yes	607 (66.3)
No	308 (33.7)
10. Who do you think should afford social oocyte freezing?	
By the individual	194 (21.2)
Private insurance	141 (15.4)
Health system	562 (61.4)
Companies and employers	18 (2)
11. Does the health system in Turkey afford social oocyte freezing except for nonmedical reasons?	
Yes	28 (3.1)
No	887 (96.9)
12. If the Turkish healthcare system had accommodated this procedure, would you have had oocyte freezing for nonmedical reasons?	
Yes	548 (59.9)
No	367 (40.1)
13. If a woman wants to freeze her eggs to preserve her fertility because she is not ready to have children, the cost of this procedure should be covered by the ministry of health.	
Yes	630 (68.9)
No	285 (31.1)
14. Would you consider donating your egg to a woman you know?	
Yes	200 (21.9)
No	285 (31.1)
15. If I decide not to use my eggs to try to get pregnant after freezing them, I would consider donating them:	
For medical research.	462 (37.7)
To help a friend or family member with fertility problems conceive a child.	201 (16.4)
An anonymous egg donor program to assist an infertile individual or couple in conceiving a child.	223 (18.2)
Other	341 (27.7)
16. What would you do if you or your partner had low ovarian reserve?	
Having children earlier than originally planned	417 (22.6)
Interrupting their education	31 (1.7)
Having IVF	390 (21.1)
Freezing oocyte or embryo	403 (21.8)
Adoption	331 (17.9)
Choosing not to have children	273 (14.9)
17. If a woman is not ready to have children in her 20 or 30 s, she should consider preserving her fertility through egg freezing.	
Strongly disagree	40 (4.4)
Disagree	77 (8.4)
Undecided	265 (29)
Agree	363 (39.7)
Strongly agree	170 (18.6)
18. Freezing eggs before age 35 can significantly prolong a woman's fertility.	
Strongly disagree	39 (4.3)
Disagree	80 (8.7)
Undecided	271 (29.6)
Agree	407 (44.5)
Strongly agree	118 (12.9)
19. If a woman's future fertility will be adversely affected by cancer treatments, she should be able to freeze her eggs to increase her chances of becoming a future mother.	
Strongly disagree	27 (3)
Disagree	20 (2.2)
Undecided	81 (8.9)
Agree	373 (40.8)
Strongly agree	414 (45.2)
20. If a woman chooses to freeze her eggs as a way of preserving her fertility before undergoing cancer treatment, the cost of this procedure should be covered by her health care services.	
Strongly disagree	27 (3)
Disagree	31 (3.4)
Undecided	91 (9.9)
Agree	302 (33)
Strongly agree	464 (50.7)
21. As part of the healthcare system, women of childbearing age should be routinely informed about egg freezing.	
Strongly disagree	24 (2.6)
Disagree	43 (4.7)
Undecided	104 (11.4)
Agree	371 (40.5)
Strongly agree	373 (40.8)
22. Should discussing the natural decline of fertility with age be part of the patient's annual checkup with a gynecologist?	
Strongly disagree	16 (1.7)
Disagree	31 (3.4)
Undecided	120 (13.1)
Agree	442 (48.3)
Strongly agree	306 (33.4)
23. I consider freezing my eggs if:	
I have not yet found a suitable partner with whom I can have children.	478 (18.9)
I am not ready to have children.	502 (19.9)
My partner is not ready to have children.	382 (15.1)
I am facing cancer treatments that could harm my future fertility.	773 (30.6)
I will prioritize my education and career plans.	390 (15.5)
24. Select factors that may influence your decision regarding oocyte freezing and fertility preservation	
Possible health risks for a child conceived using previously frozen eggs	492 (12.7)
The long-term risks to my health of my future fertility from the egg collection process	487 (12.5)
Transaction costs	487 (12.5)
Current success rates and probability of obtaining a viable pregnancy	464 (12)
Possible discomfort or side effects due to hormones and aggregation	542 (14)
How many times will I have to go through the procedure	353 (9.1)
Concerns about having to decide what to do with unused eggs	370 (9.5)
Concerns about interfering with the natural reproductive lifespan	359 (9.3)
Ethical or moral concerns regarding fertility preservation in general	227 (5.9)
Worries about negative judgments of others	94 (2.5)
25. Select factors that might influence your decision about oocyte freezing if faced with a cancer diagnosis.	
If I am in a patient relationship, my partner's feelings	224 (5.3)
Concerns about the effects of hormones or the egg retrieval procedure on my health	505 (11.9)
My prediction for full recovery	414 (9.7)
Advice from my oncology team	597 (14.1)
The importance of being a future mother	436 (10.3)
The severity of my cancer	605 (14.2)
How quickly the egg freezing process can be done/completed	314 (7.4)
Whether I have had a child before	534 (12.6)
Emotional support from my family and friends	285 (6.7)
Cost of the transaction	334 (7.8)
26. How much does a cycle egg freezing cost in Turkey, including drugs?	
10,000–20000	99 (10.8)
20,000–25000	211 (23.1)
25,000–30000	253 (27.7)
> 30,000	352 (38.5)
27. I would prefer to work for a company that has a benefit package that includes the cost of egg freezing.	
Yes	602 (65.8)
No	313 (34.2)
28. If I cannot afford to freeze my eggs, I would consider accepting money from a parent or family member for this procedure.	
Yes	584 (63.8)
No	331 (36.2)
29. The egg freezing procedure can pose risks to a woman's health and future fertility.	
Yes	340 (37.2)
No	575 (62.8)
30. Hormone injections required to freeze eggs do not have any side effects.	
Yes	192 (21)
No	723 (79)
31. In achieving a healthy pregnancy, a woman's age at the time her eggs are frozen is more important than the age at which she chooses to conceive with frozen eggs.	
Yes	639 (69.8)
No	276 (30.2)
32. A woman can successfully and safely use frozen eggs while still fertile to try to get pregnant in her 40 s and 50s.	
Yes	680 (74.3)
No	235 (25.7)
33. Most frozen eggs can be used for pregnancy by fertilizing and becoming embryos without being damaged by the thawing process.	
Yes	588 (64.3)
No	327 (35.7)
34. Long-term health effects for children born from using frozen eggs are currently unknown.	
Yes	771 (84.3)
No	144 (15.7)

**Table 4:** Comparison of the knowledge levels of women who think and do not want to have oocyte freezing.

Questions	The number of correct answers to the question in those who do not think of POC⁣^∗^ (*N* = 464), *n* (%)	The number of correct answers to the question in those who think of POC⁣^∗^ (*N* = 451), *n* (%)	*p*
1. Fertility of women at different ages?	188 (40.5)	197 (43.7)	0.333
2. What is a woman's percent chance of spontaneous pregnancy?	108 (23.3)	81 (18)	0.047
3. What is a woman's percent chance of conceiving with IVF?	121 (26.1)	138 (30.6)	0.129
4. What is a woman's percent chance of conceiving with frozen oocytes?	116 (25)	110 (24.4)	0.831
5. Which age group is best suited for freezing oocyte for nonmedical reasons?	214 (46.1)	239 (53)	0.038
6. At what age do women have a slight decrease in their ability to conceive?	117 (25.2)	156 (34.6)	0.002
7. At what age does a woman's ability to become pregnant decrease significantly?	89 (19.2)	116 (25.7)	0.018
8. For women over 30 years of age, overall health and fitness is a better predictor of fertility than age.	155 (33.4)	153 (33.9)	0.868
9. The health system in Turkey affords social oocyte freezing except for nonmedical reasons.	446 (96.1)	441 (97.8)	0.144
10. Freezing eggs before age 35 can significantly prolong a woman's fertility.	229 (49.4)	296 (65.6)	< 0.001
11. Like IVF, egg freezing requires injection of hormones and surgical removal of eggs from a woman's ovaries to stimulate egg production.	293 (63.1)	314 (69.6)	0.038
12. One treatment cycle is usually sufficient to obtain enough eggs for freezing.	225 (48.5)	204 (45.2)	0.323
13. One cycle egg freezing cost in Turkey, including drugs?	136 (29.3)	117 (25.9)	0.255
14. The egg freezing procedure may pose a risk to a woman's health and future fertility.	265 (57.1)	310 (68.7)	< 0.001
15. Hormone injections required for egg freezing have no side effects.	87 (18.8)	105 (23.3)	0.092
16. In achieving a healthy pregnancy, the age of the woman at the time her eggs are frozen is more important than the age at which she chooses to conceive with frozen eggs.	328 (70.7)	311 (69)	0.568
17. A woman in her 40 s and 50 s can successfully and safely use frozen eggs while still fertile to try to get pregnant.	324 (69.8)	356 (78.9)	0.002
18. Most frozen eggs can be fertilized and become embryos for pregnancy without being damaged by the thawing process.	286 (61.6)	302 (67)	0.093
19. Long-term health effects for children born from using frozen eggs are currently unknown.	398 (85.8)	373 (82.7)	0.202

⁣^∗^Planned oocyte cryopreservation.

## Data Availability

All data generated or analyzed during this study are included in this article. Further inquiries can be directed to the corresponding author. The individual questionnaire forms cannot be shared due to legal restrictions.
